# Effects of enhanced external counterpulsation on anginal symptoms and improvements in objective measures of myocardial ischaemia

**Published:** 2007-07

**Authors:** M Yavari, HR Montazeri

**Affiliations:** Isfahan University of Medical Sciences, Isfahan, Iran; Department of Cardiology, Shahid Chamran Hospital, Isfahan, Iran

## Abstract

**Background:**

Enhanced external counterpulsation (EECP) is a novel, potentially beneficial adjunct therapy used for angina pectoris. We assessed the efficacy of this method in relieving angina and improving objective measures of myocardial ischaemia.

**Methods:**

All patients (67) who referred for EECP to Shahid Chamran Hospital, Isfahan, Iran from 2002 to 2005 were included. Demographic data, coronary artery disease (CAD) risk factors and baseline angiographic data were collected. Anginal symptoms, Canadian Cardiovascular Society (CCS) functional class, echocardiographic parameters (ejection fraction, left ventricular end-diastolic and end-systolic diameters) and exercise test duration before and after the treatment were compared.

**Results:**

Seventy-seven per cent of patients who had undergone EECP had a positive clinical response. Exercise test duration and CCS functional class improved after the treatment. However, EECP had no significant effect on echocardiographic parameters. Efficacy was independent of age, gender, CAD risk factors, prior CCS functional class and echocardiographic parameters. Patients without left main artery involvement and those who had at least one non-obstructed artery demonstrated a greater likelihood of improvement.

**Conclusion:**

The results of this study suggested that EECP is a safe, well tolerated, and significantly effective treatment for angina pectoris.

## Summary

Enhanced external counterpulsation (EECP) is a non-invasive, pneumatic technique that can be offered to patients with angina refractory to anti-anginal medications, who are not suitable candidates for conventional revascularisation procedures.^1^ Worldwide there is a growing body of literature with regard to this modality of treatment, which was initiated quite a number of years ago.

EECP treatment produces an acute haemodynamic effect that is similar to that produced by the invasive intra-aortic balloon pump. Three sets of cuffs on the upper thigh, lower thigh and calves of each leg are inflated with compressed air during the diastolic phase of the cardiac cycle and are deflated in early systole. This rapid inflation and deflation raises diastolic aortic pressure, increases coronary perfusion pressure, provides afterload reduction, and enhances venous return with a subsequent increase in cardiac output.^2^,^3^

Prior studies suggest a potential for EECP to enhance coronary collateralisation. The simplest mechanism by which EECP might increase collateral perfusion is by opening preformed collateral channels, either directly via increasing diastolic blood pressure and flow or indirectly via release of vasodilator mediators.^4^

In this study, we assessed the effect of EECP in relieving angina and improving the objective measures of myocardial ischaemia.

## Materials and methods

Enhanced external counterpulsation equipment was supplied by the manufacturer, Vasomedical (Westbury, New York). The equipment consists of an air compressor, a console, a treatment table and two sets of three cuffs. Before a treatment session, these cuffs were wrapped around the patient’s legs, one set on each leg. Using compressed air, pressure was applied via the cuffs to the patient’s lower extremities in a sequence synchronised with the cardiac cycle.

In early diastole, pressure was applied sequentially from the lower legs to the lower and upper thighs to propel blood back to the heart. This resulted in an increase of arterial blood pressure and retrograde aortic blood flow during diastole (diastolic augmentation). At end-diastole, air was released instantaneously from all the cuffs to remove the externally applied pressure, allowing the compressed vessels to reconform, thereby reducing vascular impedance. Blood pressure changes were monitored by finger plethysmography. Daily one- to two-hour treatment sessions were typically administered for a total treatment course of 35 hours.

## Study population and protocol

All consecutive patients undergoing EECP in Shahid Chamran Hospital Heart Centre, Isfahan, Iran from 2002 were enrolled in the study (67 patients). The protocol was approved by the Ethics Committee of the University of Isfahan. Patients’ data, which had been recorded prior to treatment, included age, gender, body mass index (BMI) (weight in kg divided by the square of height in m), past history of diabetes mellitus (DM), hypertension, hyperlipidaemia, cigarette smoking and previous angiographic data (angiographic score as one-, two- or three-vessel disease). All patients were followed weekly with questionnaires about their anginal symptoms. CCS functional class (the Canadian Cardiovascular Society functional class of angina, Table 1) before and after the EECP treatment was also recorded.

**Table 1:** CCS Grading Of Angina Effort

*Functional effort associated with angina class*	
I	Ordinary physical activity does not cause angina, such as walking or climbing stairs. Angina with strenuous or prolonged exertion at work or recreation.
II	Slight limitations of ordinary physical activity. Walking or climbing stairs rapidly. Walking uphill, walking or climbing stairs after meals, or in cold or wind, or under emotional stress, or only during the few hours after wakening. Walking more than two blocks on the level and climbing more than one flight of ordinary stairs at a normal pace and in normal conditions.
III	Marked limitation of ordinary physical activity. Walking one to two blocks on the level and climbing more than one flight in normal conditions.
IV	Inability to carry on any physical activity without discomfort. Anginal syndrome may be present at rest.

## Echocardiography and exercise test

Echocardiography was performed in all patients before and after EECP treatment. The following parameters were analysed: left ventricular end-diastolic and end-systolic diameters (LVEDD, LVESD), and left ventricular ejection fraction (LVEF).

An exercise test was performed under the same circumstances for all the patients at baseline and at the end of the treatment programme (seven weeks). A symptom-limited exercise test was performed on a treadmill. The onset of anginal pain was indicated by the patient. Exercise was terminated when patients complained of moderate chest pain. A 12-lead electrocardiogram was conducted during exercise and recovery. Heart rate (HR) and ST-segment depression at rest as well as during exercise and the recovery period were recorded. Exercise duration was also recorded.

## Statistical analysis

The two-tailed paired *t*-test was used to evaluate the significance of improvement in echocardiographic parameters (LVEF, LVESD and LVEDD), exercise test duration and CCS functional class with treatment. Multivariate analysis was performed to evaluate the effect of age, gender, DM, hypertension, hyperlipidaemia, BMI, smoking, pre-treatment angiographic score, echocardiographic parameters and before-treatment CCS functional class on response to EECP.

Multiple linear regression was performed using post-treatment change in patient symptoms (chest pain) as the dependent variable and the other factors as control variables. This test was also performed to evaluate the regression between exercise test-duration improvement and the other factors.

## Results

Sixty-seven patients were enrolled in the study. All patients had angina on entry. The mean age was 63.70 ± 9.62 (range 43−85 years). Of the 70.1% who were males, 9% had left main artery involvement, 21% had two-vessel and 79% had three-vessel coronary disease. Most (89%) patients received 35 hours of EECP treatment. However 11% of patients received less than 35 hours. EECP was chosen as treatment for diverse reasons, including angina refractory to medical or surgical therapy, patient or physician preference, poor candidate for surgery due to lack of graft material or targets, or operative risk. There was no report of deterioration in anginal symptoms during therapy.

All of the patients limited by their angina either improved their symptoms (47−77% of patients) or remained unchanged in anginal symptoms after EECP (14−23% of patients). Means and standard deviations of echocardiographic parameters, exercise test duration and CCS functional class before and after EECP treatment are shown in Table 2.

**Table 2:** Comparison Of Echocardiographic Parameters, Exercise Test Duration And CCS Functional Class Before And After EECP Treatment. Data Are Presented As Mean ± SD

	*Before EECP*	*After EECP*	p*-value*
Ejection fraction	46.48 ± 13.49	48.14 ± 16.03	NS
LVESD	22.84 ± 20.39	12.83 ± 16.07	NS
LVEDD	29.9 ± 26.20	15.72 ± 19.43	NS
Exercise test duration	5.14 ± 20.18	6.09 ± 2.40	S
CCS functional class	1.82 ± 0.65	1.391 ± 0.58	S

NS: not significant; S: significant. LVESD: left ventricular end-systolic diameter. LVEDD: left ventricular end-diastolic diameter.

After EECP, the mean ejection fraction and exercise test duration was higher and the LVESD, LVEDD and CCS functional class was lower than before treatment. Two-tailed paired *t*-test analysis shows that differences were not statistically significant in ejection fraction, LVESD and LVEDD before and after EECP (*p* > 0.05), however there were significant differences between the exercise test duration (*p* = 0.014) and CCS functional class (*p* = 0.005) before and after EECP treatment.

A multivariate regression analysis revealed that there were no significant differences in response to EECP based on age, gender and CAD risk factors (DM, hypertension, BMI, smoking and hyperlipidaemia). Also, echocardiographic parameters (LVEF, LVESD and LVEDD) and before-EECP CCS functional class had no significant influence on response to EECP (it was not significant for either anginal symptom or exercise test duration as dependent variable).

The likelihood of response to treatment with EECP was significantly related to whether there was left main stem involvement or not. Patients with left main stem involvement showed significantly less benefit. Also, patients who had at least one non-obstructed artery (2VD) had greater increase in exercise time after full EECP treatment.

## Discussion

Prior studies have demonstrated the utility of EECP in carefully controlled university settings. The MUST-EECP (MUlticentre STudy of Enhanced External CounterPulsation), the first and only multicentre, prospective, randomised, blinded, placebo-controlled trial on the subject, assessed the efficacy of EECP. In this trial, patients undergoing active counter-pulsation had a significant decrease in anginal episodes, but there was no significant improvement in the duration of the exercise test.^2^

Lawson *et al.* studied 50 patients with chronic stable angina and compared the extent of coronary disease with results of radionuclide stress testing after EECP. There was significant improvement in the perfusion defects after EECP and the less the coronary disease involvement, the greater the therapeutic benefit from EECP.^3^

Another study analysed the data from an EECP registry examining effect of diastolic augmentation on the efficacy of EECP. Patients who were younger, male, without multi-vessel coronary or non-cardiac vascular disease and non-smokers were likely to have higher diastolic augmentation and greater reduction in angina class after EECP.^5^

In a further report of data from an EECP consortium (2 284 patients), an improvement was reported in up to 74% of patients with angina undergoing EECP, by one or more CCS functional classes. The younger patients had a greater likelihood of improvement.^6^

Our study demonstrated significantly improved exercise test duration, as well as CCS functional class after EECP treatment. Women and men responded equally well to EECP. Also no significant differences were observed in response to EECP based on coronary artery disease risk factors (DM, hypertension, BMI, hyperlipidaemia and smoking), baseline angiographic score and CCS functional class before EECP treatment. However, patients who did not have the left main artery involved in angiography showed a significantly greater likelihood of benefit.

The IEPR study examined the benefit of EECP in patients with heart failure. Compared with patients without heart failure, significantly fewer patients with a history of heart failure completed the course of EECP and exacerbation of heart failure was more frequent in them, although the overall major adverse cardiac events during treatment were non-significantly different between two groups.^7^ In another study, patients with congestive heart failure who were treated with EECP showed a significant improvement in their functional capacity and quality of life.^8^

In our study we found no differences in LVESD, LVEDD and ejection fraction before and after EECP treatment. These results may suggest wider opportunities for EECP use in patients with heart failure. However, we await the results of larger ongoing trials investigating the efficacy and safety of EECP in coronary heart failure patients before this treatment can be definitively recommended for treatment of this patient population.

Chronic angina impacts on the utilisation of healthcare resources, requiring repeated hospitalisations and revascularisations, so it is one of the major objectives of CAD treatment. Our study demonstrated that EECP is an emerging treatment alternative for anginal symptoms that is both safe and effective in improving angina. In current practice it is often used when angina is refractory to medical or surgical treatment, though results suggest wider opportunities for its use.

One limitation to our study was that the placebo effect of the device could not be ruled out.

In conclusion, the present study confirms EECP as a safe and practical treatment for angina in general clinical practice. Its more widespread availability as a treatment alternative may prove beneficial to patient care.
